# Impairments of social interaction in a valproic acid model in mice

**DOI:** 10.3389/fnbeh.2024.1430267

**Published:** 2024-08-29

**Authors:** Masatoshi Ukezono, Yoshiyuki Kasahara, Chihiro Yoshida, Yuki Murakami, Takashi Okada, Yuji Takano

**Affiliations:** ^1^Department of Developmental Disorders, National Center of Neurology and Psychiatry, National Institute of Mental Health, Kodaira, Japan; ^2^Department of Maternal and Fetal Therapeutics, Tohoku University Graduate School of Medicine, Sendai, Japan; ^3^Department of Hygiene and Public Health, Kansai Medical University, Hirakata, Japan; ^4^School of Psychological Sciences, University of Human Environments, Matsuyama, Japan

**Keywords:** valproic acid model, autism spectrum disorder, reaching, sniffing behavior, open field, mice

## Abstract

**Background:**

A rodent autism spectrum disorder (ASD) model based on prenatal exposure to valproic acid (VPA) is widely recognized as a prominent model. Social behavior in rodent ASD models has primarily been evaluated through a three-chamber approach test. However, in this study, we focused on social attention in the VPA model of ASD.

**Methods:**

In male C57BL/6 J mice, attentional behaviors toward conspecifics were examined through reaching tasks around 9–11 weeks of age. On embryonic day 12.5, pregnant mice underwent a subcutaneous injection of 600 mg/kg VPA sodium salt dissolved in 0.9% saline solution (VPA group) or saline solution alone (Sal group) into their neck fat. Thirty-six mice—nine each in the VPA and saline groups, and 18 partners—underwent training in reaching behavior. Subsequently, we examined whether the VPA or Sal group demonstrated focused attention toward their partners during reaching tasks. A two-way analysis of variance (ANOVA) (condition [VPA/Sal] × situation [face-to-face (attention)/not paying attention (not attention)]) was conducted on the average success rate of the situation. Additionally, we measured the duration of sniffing behavior between pairs of mice in an open field twice in total at 4 and 8 weeks of age before reaching task. The pairs were constructed by pairing a VPA or Sal group mouse with its partner, with the objective of facilitating initial encounters between the mice. A one-way ANOVA was conducted on the average duration of sniffing behavior data from 4 weeks and a second one-way ANOVA on data from 8 weeks.

**Results:**

The analysis revealed a significant interaction between condition and situation in the reaching task [*F* (1, 28) = 6.75, *p* = 0.015, η_p_^2^ = 0.19]. The simple main effect test exhibited that the “not paying attention” rate was significantly higher than that of the “face-to-face” in the VPA group (*p* < 0.01). The results revealed a not significant difference in the average duration of sniffing behavior at 4 weeks [*F* (3, 32) = 2.71, *p* = 0.06, *n.s.*, η_p_^2^ = 0.20], but significant difference at 8 weeks [*F* (3, 32) = 4.12, *p* < 0.05, η_p_^2^ = 0.28]. Multiple comparisons using the Bonferroni method revealed significant differences in the sniffing duration at 8 weeks between from the partner toward the VPA mouse and from the partner toward the Sal mouse (*p* < 0.05).

**Conclusion:**

The VPA rodent model of ASD exhibited differences in social attention compared to the saline group. By focusing on social attention and exploring various ASD models, insights can be gained from the neural mechanisms underlying gaze abnormalities during social interaction in individuals with ASD.

## 1 Introduction

Autism spectrum disorder (ASD) is characterized by two primary behavioral features: impaired social communication and restricted repetitive patterns of behavior, interest, and activity (American Psychiatric Association, 2022; Miles, 2011). It is a complex disorder influenced by various genetic and environmental factors, with most cases classified as idiopathic, meaning the cause is unknown (Karimi et al., 2017). Establishing biologically relevant animal models of idiopathic ASD presents a significant challenge (Pelch et al., 2019). Consequently, prior studies have focused on models involving prenatal exposure to valproic acid (VPA; Nicolini and Fahnestock, 2018). VPA, commonly utilized as an antiepileptic and mood stabilizer, has been linked to an increased risk of ASD when consumed during pregnancy (Chaliha et al., 2020). Epidemiological studies have exhibited a significant (4.42%) increase in ASD risk associated with VPA ingestion during pregnancy (Christensen et al., 2013). Administration of VPA during pregnancy results in offspring exhibiting behaviors indicative of ASD in rodents as well as in humans (Chaliha et al., 2020). Considering the interspecies homology observed between humans and rodents in prior studies, the VPA model is recognized as a valid animal model of idiopathic ASD (Nicolini and Fahnestock, 2018).

A three-chamber test primarily evaluates ASD-like behaviors in rodents (Murakami et al., 2021; Nicolini and Fahnestock, 2018; Sakakibara et al., 2014); it involves providing a room with a confined cage containing another conspecific and a room with an empty cage, and measuring the time spent approaching the caged conspecific as a social behavior. If the time spent approaching an empty cage does not differ from the time spent with conspecifics, it indicates ASD-like behavior and is indicative of an ASD model (Chaliha et al., 2020). In the VPA model of ASD among rats, it has been reported that a single administration of 600 mg/kg VPA on day 12.5 of pregnancy causes the offspring to consistently approach an empty cage (Schneider and Przewłocki, 2005). The VPA model of ASD in mice has also been generated using the same method mentioned above (Nicolini and Fahnestock, 2018). According to a systematic review (Chaliha et al., 2020), administration of 300–600 mg/kg VPA subcutaneously or intraperitoneally between days 9.5 and 15 of pregnant rodents increases the preference for an empty cage over other conspecifics. Administration of the same amount of VPA from days 10 to 13 of pregnancy decreases the approach to novel conspecifics in rodents, causing an impairment in social memory. Furthermore, an ultrasonic vocalization emission by pups was reduced (both frequency and/or duration) (Zhang et al., 2012; Zhang et al., 2019), and social interaction in terms of the duration of sniffing was impaired when 400–600 mg/kg VPA was administered to them (Wu et al., 2017) These effects were dose-dependent, with the highest dose of 600 mg/kg being the most effective (Chaliha et al., 2020).

A previous study investigated the autonomic nervous system activity in VPA model mice at each developmental stage prior to birth, unveiling its correlation with ASD symptoms (Kasahara et al., 2020). The autonomic nervous system activity in VPA model mice was suggested to change from the fetal period, and its evaluation during early development exhibited promise in understanding ASD. The VPA model fetuses showed decreased sympathetic activity, which may be one of the early features of the disease, as children with ASD may show changes in autonomic nervous activity (Harder et al., 2016). Because of the suggested relationship between autonomic activity and ASD symptoms or response to medication (Thapa et al., 2021), it is very important to detect early ASD symptoms through changes in autonomic activity and to study its relationship with ASD symptoms. However, previous studies focused on autonomic nervous system activity and examined the fetal period and stress exposure *in vivo* mice without actively investigating social behavioral aspects in VPA model mice (Kasahara et al., 2020; Widatalla et al., 2021). As a manipulation check to determine whether they were ASD models, only a three-chamber test was conducted.

In the three-chamber approach test, it is evident that mice cannot distinguish between conspecifics and objects by their approaching behavior. However, other aspects of sociability such as social attention, social interaction, and ultrasonic vocalization that are distinct from the approach behavior were not investigated in the prior study (Kasahara et al., 2020). Therefore, the present study explored other aspects of sociability, specifically focusing on social attention. ASD in human has been associated with a lack of eye contact and gaze during communication (Falck-Ytter et al., 2023; Griffin and Scherf, 2020; Wang et al., 2023). In mice, attentional behaviors toward conspecifics have been reported during reaching tasks that involve a series of movements to grasp and transport food to the mouth (Ukezono and Takano, 2021a, 2021b). The present study investigated attentional behaviors toward conspecifics performing reaching tasks utilizing the VPA model. Since this is a novel evaluation, experiments were conducted only on male mice, which are robust and have been used in abundant prior research. In female VPA model mice, repetitive behaviors and vocalization time under stress were reportedly reduced, but robust impairments in social behavior, as observed in the male models, were not seen (Schneider et al., 2008). Additionally, to examine social interactions between freely behaving pairs, the study recorded the behaviors of two mice in an open field and compared the frequency of social behaviors in terms of duration of sniffing between the VPA model and control groups.

## 2 Method

### 2.1 Animals

All handling and experimental procedures were conducted in accordance with the Guidelines for the Care and Laboratory Animals of Tohoku University Graduate School of Medicine and were approved by the Committee on Animal Experiments at Tohoku University (2017MdA-334).

In all studies, the C57BL/6 J mice (CLEA, Japan) were utilized. They were housed socially (4–5 mice in the same cage) in same-sex groups and in the same treatment groups in a temperature-controlled environment under a 12 h/12 h light/dark cycle (lights on at 08:00, lights off at 20:00), with food and water available *ad libitum*.

### 2.2 Prenatal VPA treatment

Female mice (7–19 weeks old) were mated with male mice of the same age range in the evening and checked for the presence of a vaginal plug the following morning. Embryonic day 0.5 (E0.5) was considered. On E12.5, 600 mg/kg VPA sodium salt (Sigma, St. Louis, MO, United States) dissolved in 0.9% saline solution (Otsuka Pharmaceutical, Tokyo, Japan) (VPA group) or saline solution alone (as a control; Sal group) was injected subcutaneously into the neck fat of the three for VPA and two for Sal pregnant mice, respectively (Kasahara et al., 2020; Roullet et al., 2013). Each solution was administered at a dose of 100 μL per 10 g of mouse body weight. From three mother mice administered VPA, total 22 pups were born and the average number of male mice per litter was 3. Out of these pups, eight died, and of the remaining 14, nine were males. From two mother mice administered saline, total 15 pups were born, of which nine were males. The average number of male mice per litter was 4.5. The average birth weight (P0) of the 22 pups from the VPA group was 1.0 g and the 15 pups from the saline group was 1.3 g.

Post birth, male and female mice were housed together with their mother until weaning. At 4 weeks, only male mice were selected and housed in the same cage. Nine mice each from the VPA and Sal groups were assigned for the reaching task. Additionally, before reaching task, they were conducted the open field test. For each behavioral test, age-matched and equally numbered mice were prepared as partners and placed in separate cages with a maximum of four or five mice per cage. In the open field test, the same nine mice each from the VPA group and the Sal group that were used in the reaching task. They were tested the open field at 4 weeks and 8 weeks of age before the reaching task. The same 18 partners were prepared at 4 weeks of age, and then at 8 weeks of age, these partners were changed so that each encounter was with a novel partner across the three behavioral tests. The three-chamber approach test was conducted and the results were consistent with prior studies (Kasahara et al., 2020).

### 2.3 Reaching test

Previous studies have demonstrated that, after the mice learn the reaching behavior, they exhibit direct attention to conspecifics’ reaching actions (Ukezono and Takano, 2021a, 2021b). Therefore, in this study, both the VPA and Sal-treated mice were trained in reaching behavior, including their partners. All aspects, including the apparatus, learning schedule, and test session procedures were consistent with those of prior studies (Ukezono and Takano, 2021a, 2021b). The mice in this experiment were housed in groups of four or five per cage, with separate cages designated for the VPA, Sal, and partner groups. It has been previously established that serving as partners leads to attention toward the reaching actions of conspecifics, despite not being cage mates (Ukezono and Takano, 2021a, 2021b).

#### 2.3.1 Apparatus

The apparatus included a reaching room and an observation room. Both compartments were 10 cm deep × 19 cm wide × 20 cm high and made of transparent acrylic, with a feeding table between the two sides. In the reaching room, a slit (10 mm) was made near the feeding table to enable mice to reach and grasp a piece of pasta. In addition, a slit (1 mm) facing the feeding table was made in the observation room. A stick was utilized to hold the pasta, intentionally placed in front of the slits by the researchers. Two video cameras were placed above and in front of the apparatus, recording the behavior of the mice (60 fps).

#### 2.3.2 Training

After the mice attained 8 weeks of age and open-field test, training for the reaching task commenced at approximately 9–11 weeks of age. Approximately 1–2 days prior to the first training session, the mice were provided the pasta and habituated. Each pasta (Spaghetti 1.8 mm n.5500 g: Barilla Japan, Chiyoda-ku, Tokyo) was approximately 250 mm long and weighed about 1 g. The pasta was cut to a length of approximately 2–3 mm and the total weighed 10 mg at a time. The mice in the VPA, Sal, and partner groups were trained twice daily in the reaching room. Twenty rewards were provided to the mice for accurately performing the act of reaching for and grasping food in a session, with each session lasting a maximum duration of 20 min. The inter-trial interval in a session depended on individual mouse behavior. In the first and second sessions, the mice were trained to reach and grasp the pasta with their forepaws. In the third or fourth session, the experimenter did not present pasta when the mouse was sitting in front of the slit but did so when the mouse was situated away from the slit. This resulted in the mice reaching for the pasta after completely turning on the spot. Additionally, the movement prior to reaching can be standardized. In the 5th–7th sessions, the mice were trained to reach and hold the pasta after ensuring that all the mice had rotated. Two mice were excluded from the Sal group. A mouse turned on the spot prior to reaching and dropped to the pasta after 10 (or more) out of 20 attempts. This conduct was considered incomplete learning. Another mouse was excluded due to an accident in the cage. Therefore, the resulting numbers of mice in the reaching behavior test were nine in the VPA group, seven in the Sal group, and 16 in the partner mice.

#### 2.3.3 Reaching behavior test

The day after the seventh session and the next day, tests were conducted to determine whether the mice in the observation room paid attention to the mice in the reaching room. During the test session, mice were allowed to have up to 20 reaching attempts. We measured whether the mice in the observation room directed their attention to the mice in the reaching room when they performed reaching actions. The pair of observers and reaching mice in the two observation tests were counterbalanced. Five pairs in the VPA group and four pairs in the Sal group underwent an observation test in the observation room post the seventh session, during which the partner performed reaching in reaching room. The remaining pairs involved partners undergoing the observation test in the observation room, whereas both the VPA and Sal groups performed reaching in the reaching room. The next day, the roles were reversed, with opposite roles executed for counterbalancing.

The situations of social attention were classified into two categories. The first was “face-to-face” based on the two heads being in a straight line through the slits by checking two cameras. We judged the situation to be “face-to-face” only when the observer was right in front of the slit. Therefore, we assumed that the observer watched the actions of the mice in “face-to-face” situations. The second situation was termed as “not paying attention,” in which the head of the observer was positioned ≥90° away from that of the reaching subject. The number of “face-to-face” and “not paying attention” instances were counted during the test session. Furthermore, the time that the mice spent in the observation room close to the slit of the test session was calculated utilizing the upper camera. We divided the observation room viewed from the upper camera into half and defined the side near the slit as “close to the slit.” We measured the time spent in this defined area using a stopwatch. Moreover, the time required to complete a single spin prior to the reaching action was measured. The starting point was the first frame in the video (60 fps) in which the mice started spinning after sitting in front of the slit and the ending point was the frame after finishing rotating and sitting in front of the slit. We measured the time with a stopwatch.

#### 2.3.4 Statistical analysis

All statistical analyses were conducted using the statistical analysis software SPSS (Statistics25: IBM Japan, Chuo-ku, Tokyo), and a risk rate of less than 5% was considered a significant difference. The percentage of trials in which the mice did not drop the pasta was calculated as the success rate during the training sessions. A one-way analysis of variance (ANOVA) was conducted to ascertain differences in success rates between conditions. We examined the frequency of “face-to-face” and “not paying attention” between the conditions. The rate of occurrence of “face-to-face” or “not paying attention” was calculated based on the number of trials for each situation divided by 20 (the total number of trials). A two-way ANOVA (condition [VPA/Sal] × situation [face-to-face/not paying attention]) was conducted on the average rate to examine whether the VPA group decreased the occurrence of face-to-face. Furthermore, we calculated the percentage of time spent in “close to the slit” in the observational room divided by the total duration of time for each test session, and compared the VPA and Sal groups using a t-test. We measured the spin time before the reaching behavior. In the experiment, the starting point was the first frame in the video (60 fps) in which the mice started spinning after sitting in front of the slit and the ending point was the frame after finishing rotating and sitting in front of the slit. We measured the time with a stopwatch and compared the average spin times between the individual and with partner situations. A two-way repeated ANOVA (condition [VPA/Sal] × situation [individual/with partner]) was conducted on the average spin speed.

### 2.4 Open field test

To measure social interactions with unfamiliar, non-cage mate conspecifics, pairs of the Sal and naïve mice, and of the VPA and partner mice, were allowed to freely explore an open field for 10 min. A total of 36 mice before reaching task were utilized in the open field test, with nine mice each from the VPA and Sal groups, and 18 partner groups. The pairs were constructed with the objective of facilitating initial encounters between the mice. Open-field tests were conducted at 4 and 8 weeks of age.

The apparatus utilized in this study was constructed entirely from gray acrylic and measured 51 cm in depth × 34 cm in width × 22 cm in height. The interior of the apparatus was designed to enable free movement.

Paired subjects were placed in the corner of the open field along the diagonal axis for 10 min. They were allowed to engage in free exploration. The interaction time during this period was subsequently measured. Pairs formed at 4 weeks of age were distinct from those formed at 8 weeks of age, with different naïve partners selected for each pairing.

The duration of sniffing, which is recognized as a behavior indicative of social interaction (Wesson, 2013), was measured with a stopwatch from the videos recorded by the video cameras. Sniffing behavior was individually quantified to assess how much each subject engaged in it. Specifically, the duration for which the mouse’s nose made contact with any part of its partner’s body was recorded. Furthermore, the duration of sniffing from the mice in the VPA and Sal groups toward naïve partner mice, as well as sniffing from naïve partner mice toward the VPA and Sal groups, were calculated separately. A one-way ANOVA was conducted on the average duration of sniffing behavior at four and 8 weeks.

## 3 Results

The frequent reports of immaturity resulting from VPA treatment (Kotajima-Murakami et al., 2019; Roullet et al., 2010), this experiment also examined the differences in body weight at 4 and 10–12 weeks of age to investigate physical maturation. The body weight at 4 weeks of age was at the start of the open field test, and the body weight at 10–12 weeks of age was at the end of the reaching task. The average body weights were as follows: VPA in 4 weeks, 13.81 g (SD = 3.05); VPA in 10–12 weeks, 21.7 g (SD = 1.49 g); Sal in 4 weeks, 12.84 g (SD = 2.8 g); and Sal in 10–12 weeks, 25.36 g (SD = 2.3 g). A two-way repeated ANOVA (condition [VPA/Sal] × age [4 weeks/10–12 weeks]) was conducted on the average body weight. The results revealed a no main effect of condition [*F* (1, 15) = 1.19, *p* = 0.29, *n.s.*, η_p_^2^ = 0.073], significant main effect of age [*F* (1, 15) = 375.25, *p* = 0.000, η_p_^2^ = 0.96], and significant interaction of condition and age [*F* (1, 15) = 19.92, *p* = 0.000, η_p_^2^ = 0.57]. The results of multiple comparison using the Bonferroni method revealed the difference in body weight between the Sal and VPA groups at “4 weeks” was not significant (*p* = 0.51), but there was a significant difference between the Sal and VPA groups at 10–12 weeks (*p* < 0.01).

In the reaching test, during the seventh training session, the average success rates for reaching were as follows: VPA, 77.22% (SD = 8.2); Sal, 74.29% (SD = 4.95); and naïve partner, 80% (SD = 5.3). A one-way ANOVA was conducted to ascertain differences in success rates. The results indicated no significant differences [*F* (2, 29) = 1.96, *p* = 0.16, *n.s.*, η_p_^2^ = 0.12]. In contrast to Ukezono and Takano’s (2021a, 2021b) study, learning curves were not calculated because of subjects’ learning rotations extending from Sessions 4 to 6. To facilitate rotation learning, food rewards were provided, making it challenging to calculate the success rates of the reaching actions after rotation. Excluded subjects included one in the VPA group and three in the Sal group, each with success rates falling below 50% in Session 7 and not fully learning rotations.

**Figure 1:**
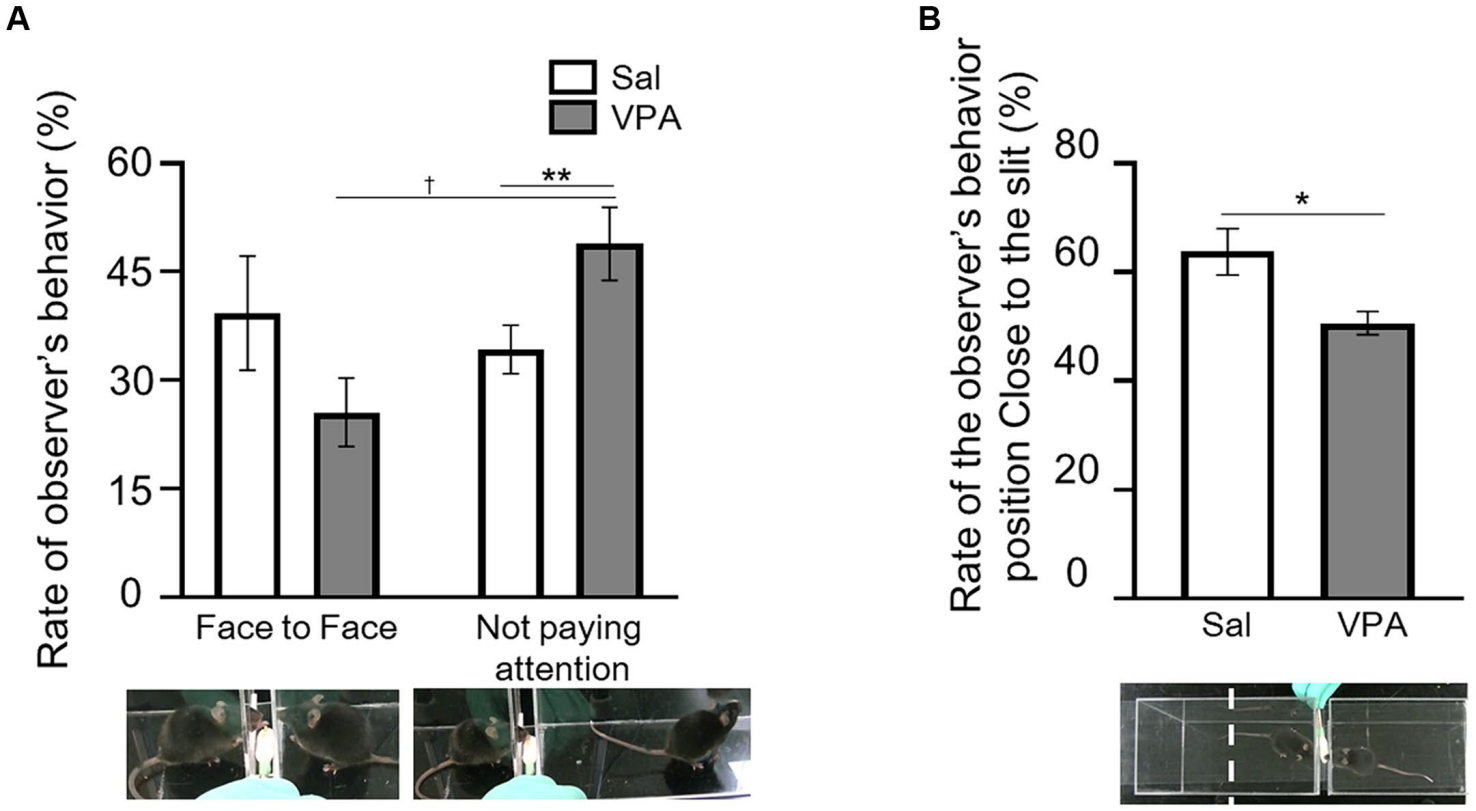
Behavior during observation of reaching by partner. **(A)** Differences in the occurrence rate of “face-to-face” and “not paying attention” situations between the Sal and VPA groups. White bars represent data from the Sal group and gray bars represent data from the VPA group. Under the pictures are the sample of face-to-face and not paying attention. **(B)** Differences in the position of the observer in the observation room depending on whether the mouse in the observation room is from the Sal or VPA group. Error bars represent the standard error of the mean. ^**^p < 0.01, ^*^p < 0.05, ^†^*p* < 0.10.

The rate of occurrence was calculated based on the number of trials with the situations of “face-to-face” and “not paying attention,” which constituted the total number of trials in a session (Figure 1A). The mean occurrence rate of the “face-to-face” situation in the VPA group was 25.6% (SD = 14.24), and that of “not paying attention” was 48.9% (SD = 15.16). In the Sal group, the mean occurrence rate of the “face-to-face” situation was 39.3% (SD = 20.9), and that of “not paying attention” was 34.3% (SD = 8.86). A two-way ANOVA (condition [VPA/Sal] × situation [face-to-face/not paying attention]) was conducted on the average rate. The results revealed an interaction of condition and situation [*F* (1, 28) = 6.75, *p* = 0.015, η_p_^2^ = 0.19], but no main effect of either [condition: *F* (1, 28) = 0.006, *p* = 0.94, *n.s.*, η_p_^2^ = 0; situation: *F* (1, 28) = 2.83, *p* = 0.10, η_p_^2^ = 0.09]. The results of the simple main effect test revealed that the frequency of “not paying attention” was significantly higher than that of the “face-to-face” situation in the VPA group (*p* < 0.01). Additionally, there was a significant rate of the “not paying attention” between the VPA (mean = 48.9%) and Sal (mean = 34.3%) groups (*p* = 0.069). For the manipulation check, the mean occurrence rate of the “face-to-face” situation of the partners was calculated and compared with the Sal group using a t-test. The results revealed no difference between the Sal group and the partner [*t* (9) = 0.19, *p* = 0.85, *n.s.*, *d* = 0.11]. We calculated the percentage of time spent in close to the slit in the observational room, relative to the total duration of each test session (Figure 1B), and compared the VPA and Sal groups using a t-test. The results revealed a significant difference between the groups [*t* (9) = 2.74, *p* < 0.05, *d* = 1.38].

**Figure 2:**
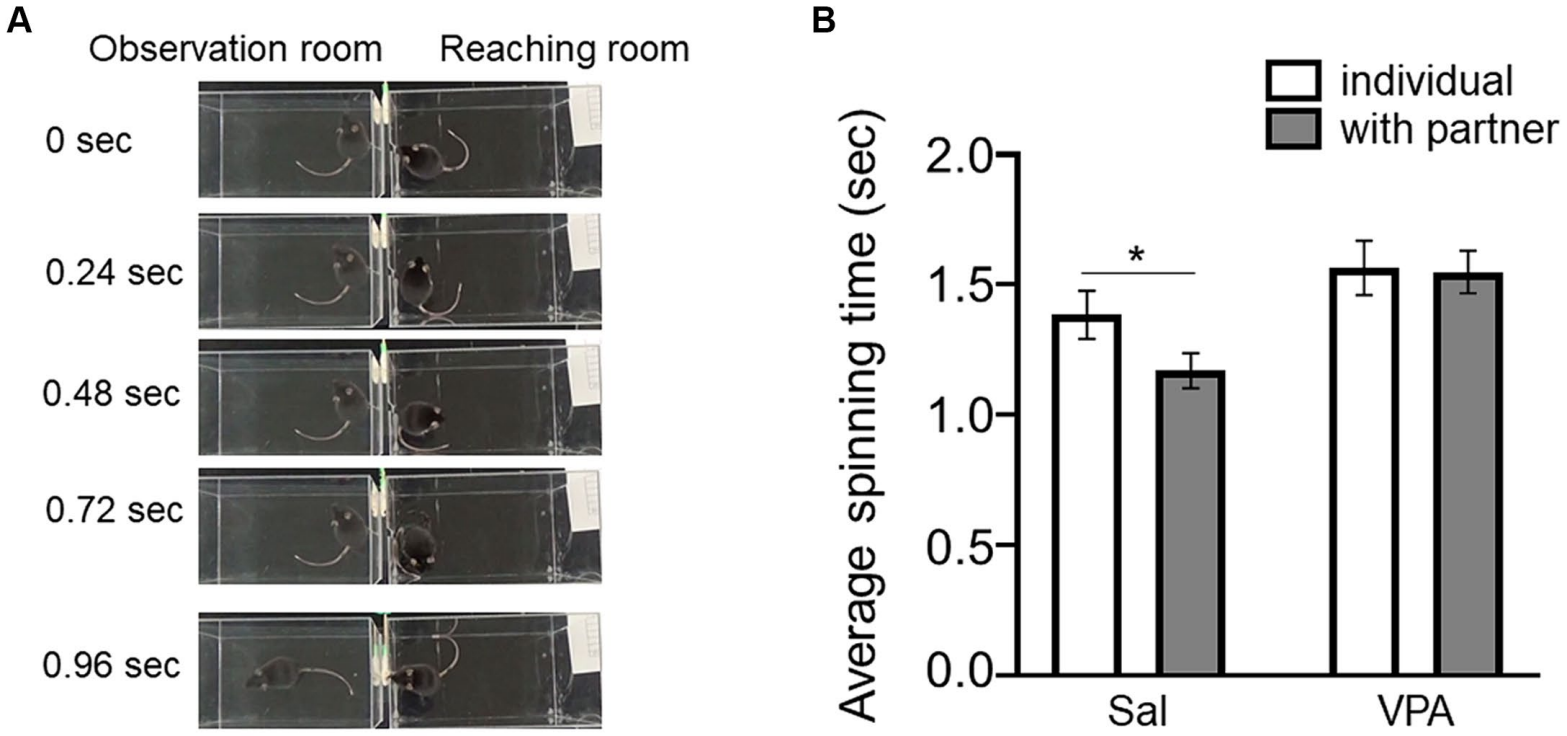
Effects of the observation of other conspecifics on spin time prior to the reaching behavior. **(A)** Sample pictures of spin behavior prior to reaching. **(B)** Difference in spin time depending on whether there is an observer (Sal group or VPA group). Error bars represent standard error of the mean. ^*^p < 0.05.

Subsequently, the speed of spins (Figure 2A) in reaching individuals was compared depending on the absence of observers in Session 7 in the Sal (mean = 1.38 s, SD = 0.24) and VPA (mean = 1.56 s, SD = 0.31) groups, and in the presence of a partner in the Sal (mean = 1.17 s, SD = 0.18) and VPA (mean = 1.55 s, SD = 0.24) groups (Figure 2B). A two-way repeated ANOVA (condition [VPA/Sal] × situation [individual/with partner]) was conducted on the average spin speed. The results revealed a main effect of condition [*F* (1, 14) = 6.03, *p* = 0.03, η_p_^2^ = 0.3], and significant main effect of situation [*F* (1, 14) = 3.86, *p* = 0.07, η_p_^2^ = 0.22], but no interaction of condition and situation [*F* (1, 14) = 2.85, *p* = 0.11, η_p_^2^ = 0.17]. The results of multiple comparison using the Bonferroni method revealed that the “with partner” situation in the Sal group was significantly faster than the “with partner” situation in the VPA group (*p* < 0.01) and the “individual” situation in the Sal group (*p* < 0.05). A tentative analysis for reference was conducted using a paired *t*-test on the mean spin times of partners between the absence of observers in Session 7 and the presence of the Sal group in the observation room, revealing a significant difference [*t* (9) = 3.15, *p* < 0.05, *d* = 1.19].

**Figure 3:**
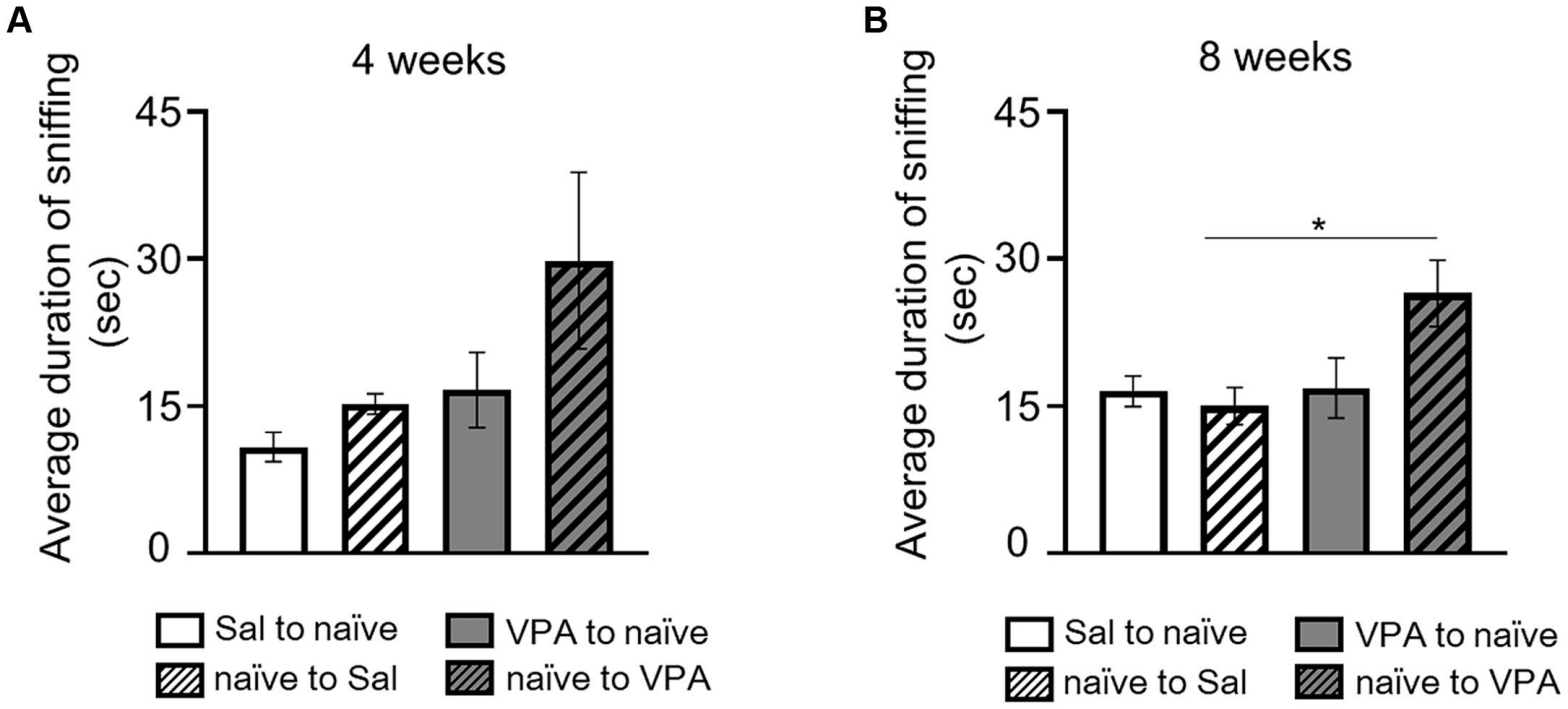
Social interaction of two mice in an open field. **(A)** Mean duration of sniffing behavior for each group at 4 weeks of age. **(B)** Mean duration of sniffing behavior for each group at 8 weeks of age. *p < 0.05.

In the open field test, at 4 weeks of age, the duration of sniffing behavior in free movement was measured for both the Sal and VPA groups, pairing them with naïve partners of the same age. The durations of sniffing from the Sal and VPA groups toward naïve partners, and from naïve partners toward the Sal and VPA groups, at 4 weeks of age, were as follows: Sal to naïve: 10.82 s (SD = 4.51); VPA to naïve: 16.63 s (SD = 11.49); naïve to Sal: 15.21 s (SD = 3.12); and naïve to VPA: 29.82 s (SD = 26.98; Figure 3A). The results of the duration of sniffing behavior at 8 weeks of age were as follows: Sal to naïve: 16.5 s (SD = 4.64); VPA to naïve: 16.83 s (SD = 9.17); naïve to Sal: 14.98 s (SD = 5.69); and naïve to VPA: 26.49 s (SD = 10.16; Figure 3B). A one-way ANOVA was conducted on the average duration of sniffing behavior at 4 weeks of age. The results revealed no significant difference [*F* (3, 32) = 2.71, *p* = 0.06, *n.s.*, η_p_^2^ = 0.20]. Another one-way ANOVA was conducted on the average duration of sniffing behavior at 8 weeks of age. The results revealed a significant difference [*F* (3, 32) = 4.12, *p* < 0.05, η_p_^2^ = 0.28]. Multiple comparisons were conducted using the Bonferroni correction. There were significant differences between the mean duration of sniffing in the naïve to VPS and naïve to Sal (*p* < 0.05) conditions, and a significant trend between naïve to VPS and other conditions (Sal to naïve: *p* = 0.06; VPA to naïve: *p* = 0.07).

## 4 Discussion

This study examined social attention behavior toward conspecifics, an aspect of sociability not measured in the three-chamber test. The VPA group exhibited a significantly higher proportion of “not paying attention” than “face-to-face” situations, as well as a higher proportion of “not paying attention” situations compared with the Sal group. The results of the training sessions demonstrated that the VPA group could learn reaching behaviors. It is unlikely that the reaching behavior of partner conspecifics was unnoticed. The average proportion of “face-to-face” situations among the partners in the Sal group did not significantly differ from that of the Sal group, indicating consistency with prior studies (Ukezono and Takano, 2021a, 2021b). The VPA group exhibited a higher proportion of “not paying attention” compared with conspecifics. Therefore, the VPA group may be less likely to exhibit social attention behavior toward conspecifics.

An important point is the high proportion of “not paying attention” exhibited by the VPA group. Research on social attention in humans with ASD has examined whether the distinctive attentional characteristics of ASD may reflect differences in motivation as opposed to attention itself (Falck-Ytter et al., 2023). The time taken close to the slit in the observational room by the VPA group was at chance level. This is consistent with the lack of differences in the approach time to objects and conspecifics in prior three-chamber approach tests (Murakami et al., 2021; Nicolini and Fahnestock, 2018; Sakakibara et al., 2014). “Not paying attention” was defined as when the head of the observer was positioned ≥90° away from that of the reaching conspecifics. Therefore, it is possible that the VPA group engaged in other motivation-based behaviors. If the lack of understanding of social signals from other conspecifics was a contributing factor to the emergence of ASD-like behaviors, one would expect a chance level of behavior without any discernible trend. Although this study suggests that the VPA group may not attend to the actions of other conspecifics, it also raises the possibility that the group may have difficulty approaching situations in which other conspecifics obtain food rewards.

Furthermore, the VPA model not only exhibited decreased attentional behavior but also failed to exhibit a facilitation in spinning speed resulting from being attended to, known as social facilitation (Ukezono et al., 2015; Zajonc, 1965). Social facilitation is a behavioral enhancement that occurs in the presence or absence of other individuals and has been robustly demonstrated in rodents and humans (Bond and Titus, 1983; Herman, 2017; Takano and Ukezono, 2014; Ukezono et al., 2015; Ukezono and Takano, 2021a, 2021b). Prior studies have also exhibited an increase in spinning speed prior to the reaching behavior (Ukezono and Takano, 2021a, 2021b). In this study, compared with the spinning speed during Session 7 in the Sal group, the spinning speed was faster when partners were present in the observation room. This finding suggests that social facilitation occurred robustly in this study. However, in the VPA group, there was no facilitation of spinning speed owing to the presence or absence of other conspecifics. This suggests that the VPA group is less influenced by the presence of conspecifics. Children with ASD have been reported to exhibit no improvement in task performance in social contexts compared with non-social contexts, suggesting the absence of social facilitation in ASD (Chevallier et al., 2014). The absence of social facilitation in another ASD mice model has also been reported (Yoshizaki et al., 2021). Considering the possibility of phenomena specifically occurring in ASD, it is possible to consider VPA animal models as valid ASD models. In the context of free interaction between two mice, the VPA group did not exhibit any differences compared with the Sal group. Specifically, focusing on sniffing behavior in an open field and measuring which subject initiated the sniffing behavior, there were no differences in the duration of sniffing. Prior research on the sociability of VPA subjects in open-field settings has exhibited a decrease in sociability compared with control conditions (Chaliha et al., 2020). Most studies have utilized pairs of VPA subjects, with a few studies utilizing pairs comprising a VPA subject and a naïve subject, and there is a lack of consistency in the results (Chaliha et al., 2020; Christensen et al., 2013; Nicolini and Fahnestock, 2018). In the reaching test, the VPA group exhibited a significantly lower proportion of approach behavior toward the slit compared to the Sal group. However, in the open field test, the VPA group showed no difference in sniffing behavior toward naïve partners compared to the Sal group at either 4 or 8 weeks of age. One possible reason for this discrepancy is that in the reaching test, conspecifics were separated by a transparent wall, whereas in the open field test, direct contact was possible. This study cannot address this issue; thus, future research should investigate the effects of contact availability on the approach behavior of VPA model animals.

The sniffing behavior toward the VPA group by naïve partners was significantly longer than that toward the Sal group. Social behaviors in an open field are inherently the result of interactions between two mice. Therefore, the prolonged engagement of naïve partners in social behavior toward the VPA subjects is believed to be influenced by the social behavior exhibited by the VPA subject. Furthermore, at 4 weeks of age, the duration of the sniffing behavior exhibited a large standard deviation, whereas at 8 weeks of age, the standard deviation decreased, while the mean value remained almost unchanged. This suggests a variability in sociability behaviors among the VPA models at 4 weeks of age, potentially influencing the duration of sniffing behavior toward naïve partners. This variability decreased by 8 weeks of age, indicating a hypothesis worth considering. The variation at 4 weeks of age might be due to delayed sexual maturation, as previous studies have reported lower sexual maturation by VPA exposure (Snyder and Badura, 1995). This study did not include data on weight progression or measurements of sexual maturation. However, future investigations should address the effects of VPA-induced immaturity on social behaviors at 4 weeks of age. If immaturity is confirmed, conducting intervention studies at this age to examine the effects on social behavior beyond 8 weeks of age would be of significant clinical relevance.

The following limitations can be considered in this study: First, we could not comment on differences that might arise if the experiments were conducted with female mice, as they were exclusively conducted with male mice. In the VPA model of female mice, it has been reported that social behaviors differ from those of male mice (Schneider et al., 2008). Future research should investigate the effects of sex differences on social attention. The second point concerns the grip strength of VPA model mice. Since it has been reported that grip strength ability is reduced in VPA model mice (Wagner et al., 2006), it is possible that the ability to grasp objects is reduced among them. This may have affected the “pasta grabbing” behavior in this study. However, the success rate of reaching in this study was not different from the Sal group, at approximately 80%. Furthermore, previous studies of reaching behavior (Takano and Ukezono, 2014; Whishaw et al., 1991; Klein et al., 2012) have mainly used pellet tablets, with success rates of almost 65% in rats (Takano and Ukezono, 2014) and 75% in mice (Ukezono and Takano, 2021a, 2021b), which is consistent with the results of the present experiment. Therefore, in the present study, while no motor impairments were detected in the VPA model, grip strength was not measured, indicating the need for more comprehensive examination. The third point is the possibility exists that weight loss in mice administered VPA may affect learning of reaching task. As observed in previous studies (Kotajima-Murakami et al., 2019; Roullet et al., 2010), the body weight of the VPA group was significantly lower than that of the Sal group as the mice aged. It is possible that weight loss is related to a decrease in grip strength and immaturity. However, in this study, no differences were observed in the success rate of reaching compared to previous research. It was also shown that immaturity due to weight loss did not affect learning of reaching. Therefore, it is considered that the impact on this reaching task is minimal. The fourth point is the small number of mother mice. Although the mice used are C57BL/6 J, which should not present any genetic issues, the small number of mother mice could potentially have influenced the results of this study.

This study suggests that VPA mice may not attend to conspecifics, and even when attended to by others, they do not respond. In the future, by focusing on social attention and investigating other animal models of ASD, the neural mechanisms underlying gaze issues observed in individuals with ASD during social communication can be examined. Additionally, naïve conspecifics exhibited relatively prolonged sociability behaviors toward the VPA group in the open field test. The behavior of model animals has been primarily investigated in prior studies; however, this study demonstrates the importance of examining social behavior from healthy subjects to model animals. By utilizing changes in the social behavior of healthy subjects as an indicator when intervening to improve sociality in model animals, it is possible to conduct investigations that differ from those conducted previously. This study contributes to understanding sociability differences in VPA subjects compared with healthy controls and underscores the importance of considering social behavior in both healthy and model animals in ASD research.

## Data Availability

The raw data supporting the conclusions of this article will be made available by the authors, without undue reservation.
